# Use of the WHO hand hygiene self-assessment framework tool in Dutch hospitals

**DOI:** 10.1186/1753-6561-5-S6-P131

**Published:** 2011-06-29

**Authors:** A Voss, T de Ruiter, G van Knippenberg-Gordebeke, MC Vos

**Affiliations:** 1 Medical Microbiology & Infectious Diseases, Canisius-Wilhelmina Hospital, Nijmegen, Netherlands; 2 Medical Microbiology , Radboud University Nijmegen Medical Centre, Nijmegen, Netherlands; 3 Infection Control, Groene Hart Hospital, Gouda, Netherlands; 4 KNIP Consultancy Infection Prevention, Venlo, Netherlands; 5 Medical Microbiology, Erasmus Medical Centre, Rotterdam, Netherlands

## Introduction / objectives

Recently, the WHO First Global Patient Safety Challenge team launched its newest tool, the Hand Hygiene Self-Assessment Framework, a validated tool to assess hand hygiene promotion and practices in health-care facilities (http://www.who.int/gpsc/country_work/hhsa_framework/en/index.html). Aim of the present study is to access the hand hygiene status-quo of Dutch hospitals.

## Methods

A working party of members from the Dutch Society of Infection Control Practitioners and the Dutch Society for Medical Microbiology decided to use the WHO’s self-assessment as a tool to progress hand hygiene promotion in the Netherlands. The framework tool was transformed into an on-line tool (e-trinity, Belgium) that allows data collection and automatic feed-back to all participants. Clinical microbiologists and infection control practitioners were contacted with support of the professional societies.

## Results

The survey is planned for the beginning of April 2011 in order to use the data for national hand hygiene promotion on the 5^th^ of May. The working party expects a 50% response rate, thus is aiming at data from at least 50 of the 100 hospitals of the country. Individual hospitals/participants receive their score automatically via the electronic system. Members of the working party will anonymously evaluate the combined national data.

## Conclusion

The Dutch are part of a minority of countries that after signing the WHO pledge did not engage into a national campaign. In addition, a recent study showed that the compliance with the 5 moments of hand hygiene is one of the lowest internationally reported (19%). The results of the self-assessment framework tool should help to better advance future hand hygiene campaigns.

## Disclosure of interest

None declared.

